# IGF-1 mediated phosphorylation of specific IRS-1 serines in *Ames dwarf* fibroblasts is associated with longevity

**DOI:** 10.18632/oncotarget.6112

**Published:** 2015-10-14

**Authors:** John Papaconstantinou, Ching-Chyuan Hsieh

**Affiliations:** ^1^ The Department of Biochemistry and Molecular Biology, University of Texas Medical Branch, Galveston, Texas, USA

**Keywords:** IGF-1, dermal fibroblasts, Ames dwarf mouse, IRS-1 serine phosphorylation, longevity, Gerotarget

## Abstract

Insulin/IGF-1 signaling involves phosphorylation/dephosphorylation of serine/threonine or tyrosine residues of the insulin receptor substrate (IRS) proteins and is associated with hormonal control of longevity determination of certain long-lived mice. The stimulation of serine phosphorylations by IGF-1 suggests there is insulin/IGF-1 crosstalk that involves the phosphorylation of the same serine residues. By this mechanism, insulin and IGF-1 mediated phosphorylation of specific IRS-1 serines could play a role in longevity determination.

We used fibroblasts from WT and *Ames* dwarf mice to examine whether: (a) IGF-1 stimulates phosphorylation of IRS-1 serines targeted by insulin; (b) the levels of serine phosphorylation differ in WT *vs*. *Ames* fibroblasts; and (c) aging affects the levels of these serine phosphorylations which are altered in the *Ames* dwarf mutant. We have shown that IRS-1 is a substrate for IGF-1 induced phosphorylation of Ser^307^, Ser^612,^ Ser^636/639^, and ^Ser1101^; that the levels of phosphorylation of these serines are significantly lower in *Ames* vs. WT cells; that IGF-1 mediated phosphorylation of these serines increases with age in WT cells. We propose that insulin/IGF-1 cross talk and level of phosphorylation of specific IRS-1 serines may promote the *Ames* dwarf longevity phenotype.

## INTRODUCTION

The extended lifespan of the *Ames* and Snell dwarf mice is attributed to the attenuation of the insulin/IGF-1 signaling pathways [1, 2]. In these mice GH deficiency reduces production and circulating levels of insulin and IGF-1 [1, 3]. Reduced IGF-1 signaling affects insulin sensitivity suggesting that crosstalk occurs between IGF-1 and insulin signaling [4].

Control of mammalian aging by IGF-1is based on the increased longevity of hypopituitary growth hormone (GH)-deficient mice in which reduced IGF-1 expression and peripheral levels are characteristics of increased lifespan [5-7]. Subsequent studies of mice heterozygous for the IGF-1R [IGF-1R^(+/−)^] provided direct evidence that IGF-1 plays a role in controlling mouse longevity [8, 9]. Low levels of circulating IGF-1 are, therefore, a common feature of several long-lived mouse models (*Ames*, *Snell*, and *IGF-1R^(+/−)^*). Decreased mRNA and protein pool levels of the hepatic IGF-1 axis in *Ames* mice suggest that the regulation of genes targeted by the insulin/IGF-1-signaling pathway may contribute to physiological conditions supporting longevity [7]. Thus, in the *Ames* mouse, IGF-1 also regulates the insulin signaling pathway suggesting the involvement of insulin/IGF-1 crosstalk interactions.

Phosphorylation of serine/threonine (Ser/Thr) or tyrosine (Tyr) residues of the insulin receptor substrate (IRS) proteins regulate insulin signaling [10]. Phosphorylation of the IRS Ser residues inhibits Tyr phosphorylation thereby serving as a physiological negative-feedback control mechanism [11]. Insulin stimulated Ser phosphorylation observed in hyperglycemia [12] or in response to proinflammatory cytokines [13-16] suggests this as the mechanism of acute and chronic stress mediated insulin resistance [17]. Thus, the stimulation of Ser/Thr phosphorylation of IRS-1 (and IRS-2), impairs its association with the insulin receptor (IR) thereby inhibiting insulin-stimulated Tyr-phosphorylation of both IRS-1 and IR [13, 18-21]. Control of IRS-1 signaling is thus achieved by the differential phosphorylation of Ser/Thr and Tyr residues. These phosphorylations are part of the physiological processes of longevity determination as well as the development of insulin and IGF-1 resistance.

Phosphorylation of IRS-1 on Ser^307^. Ser^612^, Ser^636/639^, and Ser^1101^ negatively regulate several functions of IRS-1 which include: a) phosphorylation of Ser^309^ which uncouples IRS-1 from the insulin receptor (IR); decreases tyrosine phosphorylation and increases degradation of the IR; b) phosphorylation of Ser^612^ and Ser^636/639^ reduces the IRS-1/PI3-kinase association [11].

Although insulin and IGF-1 signaling are initiated by specific receptors there is considerable crosstalk between these pathways [4, 14, 22]. This raises the question of whether insulin/IGF-1 crosstalk involves the phosphorylation of the same IRS-1 Ser residues. By this mechanism, insulin and IGF-1 crosstalk could regulate longevity and the development of insulin and IGF-1 resistance [13].

In past studies we demonstrated that fibroblast cultures derived from young and aged *Ames* dwarf mice maintain their *in vivo* characteristics of resistance to mitochondrial generated oxidative stress [23]. Based on these observations we used these cells to address the question of whether: (a) IGF-1 stimulates the phosphorylation of the same IRS-1 Ser residues that are targeted by insulin; (b) the levels of Ser phosphorylation differ in WT vs. *Ames* dwarf fibroblasts; and (c) aging affects the levels and pattern of IGF-1 stimulated Ser phosphorylations.

We propose that the results of our experiments would provide information on the mechanism by which IGF-1 participates in the regulation of insulin-GH signaling and the determination of longevity.

## RESULTS

Multiple physiological functions, including longevity determination and insulin/IGF-1 resistance are regulated by IRS-1signaling involving phosphorylation-dephosphorylation of numerous Ser/Thr and Tyr residues. In these studies we examined whether IGF-1 stimulates the phosphorylation of IRS-1 Ser^307^, Ser^612^, Ser^636/639^ and Ser^1101^ residues that are known to be phosphorylated in response to insulin treatment, whether these IGF-1 stimulated phosphorylations in *Ames* fibroblasts are consistent with decreased insulin/IGF-1 pathway activity associated with longevity.

### IGF-1 stimulates IRS-1 Ser^307^ phosphorylation in young and aged wild type, and aged *Ames* dwarf fibroblasts

**Figure 1:**
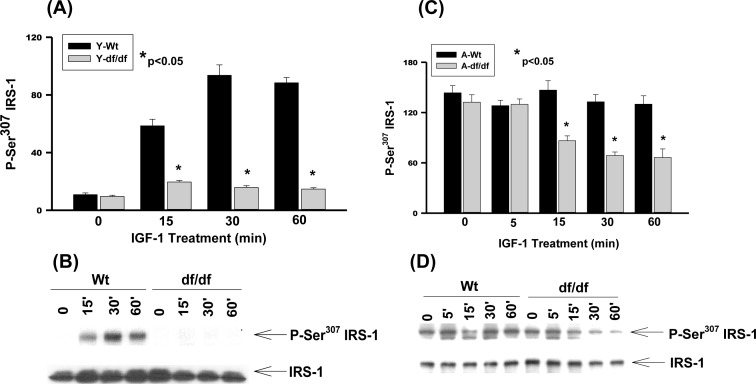
The IGF-1 stimulated phosphorylation of IRS-1 Ser^307^ in young and aged WT and *Ames* dwarf mouse tail fibroblasts **A.** A bar graph and **B.** immunoblot analysis of IRS-1 Ser^307^ phosphorylation in fibroblasts from young (3-6 mos) WT and *Ames* dwarf mice. **C.** A bar graph and **D.** immunoblot analysis of the phosphorylation of IRS-1 Ser^307^ in fibroblasts from aged (21-24 mos) WT and *Ames* dwarf mice. Statistical analyses were performed using the two-tailed *t*-test to show the mean difference between age-matched groups at a significance level of 0.050. The symbols (*) indicate statistical significance for the values represented by the bar or time point.

The longevity of *Ames* dwarf [*Prop1^(−/−)^*] mice is attributed to decreased levels of tissue and circulating insulin and IGF-1[1, 3] and the differential phosphorylation of IRS-1 Ser residues may be part of the mechanism that regulates the altered insulin/IGF-1 signaling that promotes longevity. To address this we examined whether IGF-1 stimulates phosphorylation of the same IRS-1 Ser residues that are insulin-stimulated. The responses by the young *Ames* fibroblasts and their age-matched WT controls to IGF-1 treatment indicate that Ser^307^ phosphorylation is stimulated in WT fibroblasts by ~6-fold at 15 minutes and ~10-fold at 30 minutes and remains elevated up to 60 minutes of treatment (Figure 1A and 1B). On the other hand phosphorylation of Ser^307^ is severely attenuated in the *Ames*-derived fibroblasts suggesting that these cells maintain the characteristic decreased levels of insulin/IGF-1 signaling associated with *Ames* longevity.

There is a ~14-fold increase in the endogenous level of IRS-1 Ser^307^ phosphorylation in aged WT and dwarf fibroblasts (Figure 1C, 1D). Furthermore treatment with IGF-1 did not further enhance phosphorylation of Ser^307^ in either the aged WT or *Ames* fibroblasts. Thus, in both cell types the elevated endogenous levels of Ser^307^ phosphorylation and the loss of response of IGF-1 suggest significantly altered physiological functions. The significant endogenous derepression of Ser^307^ phosphorylation in both WT and Ames untreated fibroblasts, and loss of response to IGF-1 are indicative of major alterations of metabolic functions.

Interestingly, the aged *Ames* fibroblasts show a decreased level of Ser^307^ phosphorylation at 15, 30, and 60 minutes after IGF-1 treatment suggesting that the phosphorylation is responsive whereas the WT fibroblasts are refractive in that they maintain the elevated level of Ser^307^ phosphorylation (Figure 1C and 1D). These results suggest that functions regulated and inducible by P-Ser^307^ persist in the WT controls, but are attenuated and transient in the Ames dwarf cells.

### IGF-1 stimulates IRS-1 Ser^612^ phosphorylation in young and aged wild type, and aged *Ames* dwarf fibroblasts

The G protein-coupled receptor kinase-2 (GRK2) is a Ser/Thr kinase that upon endothelin-1 (ET-1) stimulation associates with IRS-1 thereby promoting ET-1 mediated Ser^612^ phosphorylation and IRS-1 degradation [24]. GRK2 thus plays a role in chronic ET-1 induced insulin resistance by inhibiting IRS-1. Elevated GRK2 functions as a negative regulator of insulin action by interfering with G protein-q/11α subunit signaling. Thus, upon ET-1 activation, GRK2 associates with IRS-1 and promotes ET-1 mediated IRS-1 Ser^612^ phosphorylation and degradation which is associated with insulin resistance [24]. Furthermore, the phosphorylation of Ser^612^ is uniquely associated with ET-1 and GRK2 in myocardial ischemic injury [24, 25]; type 2 diabetes [26]. obesity and hypertension [26, 27].

**Figure 2:**
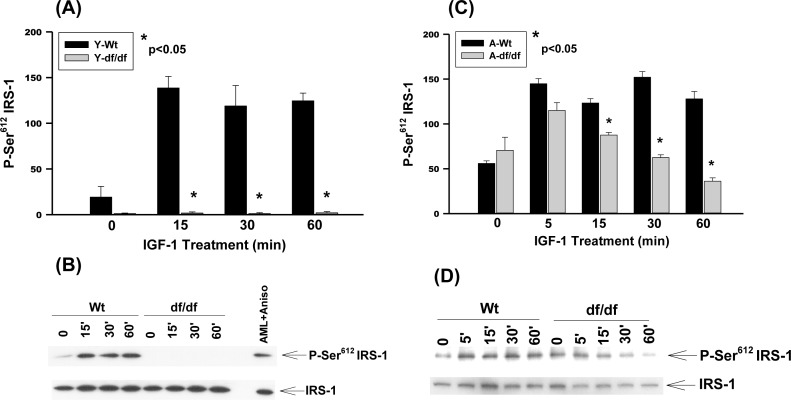
The IGF-1 stimulated phosphorylation of IRS-1 Ser^612^ in young and aged WT and *Ames* dwarf mouse tail fibroblasts **A.** A bar graph and **B.** immunoblot analysis of IRS-1 Ser^612^ phosphorylation in fibroblasts from young (3-6 mos) WT and *Ames* dwarf mice. **C.** A bar graph and **D.** immunoblot analysis of the phosphorylation of IRS-1 Ser^612^ in fibroblasts derived from aged (21-24 mos) WT and *Ames* dwarf mice. Statistical analyses were performed using the two-tailed *t*-test as described in Figure 1 and in Methods.

Our results show that IGF-1 stimulates the phosphorylation of Ser^612^ in WT fibroblasts whereas the *Ames* fibroblasts do not respond to this treatment (Figure 2A and 2B). On the other hand the level of IGF-1 stimulated Ser^612^ phosphorylation in aged WT is similar to that of the young fibroblasts whereas phosphorylation of the *Ames* fibroblasts is significantly derepressed, although the level of expression is lower than that of the WT response (Figure 2C and 2D). The endogenous levels of Ser^612^ phosphorylation are thus increased in both aged WT and *Ames* fibroblasts and IGF-1 induces further phosphorylation in both cell types. There are, however, significant differences between WT and *Ames* fibroblasts in that (a) young *Ames* fibroblasts are resistant to IGF-1 stimulated phosphorylation and (b) the inducible level of Ser^612^ phosphorylation in the aged WT is similar to that of the young cells and c) the level of induction of the WT cells remains elevated for 60 minutes, whereas in the *Ames* cells it peaks in 5 minutes and declines rapidly (Figure 2C and 2D).

### IGF-1 stimulates Ser^636/639^ phosphorylation in young and aged WT and aged *Ames* dwarf fibroblasts

**Figure 3:**
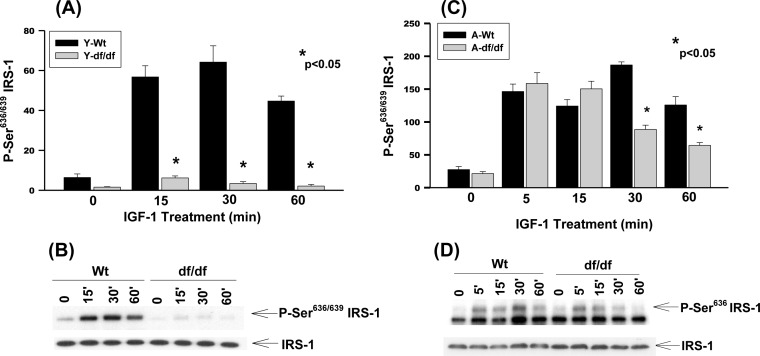
The IGF-1 stimulated phosphorylation of IRS-1 Ser^636/639^ in young and aged WT and *Ames* dwarf mouse tail fibroblasts **A.** A bar graph and **B.** immunoblot analysis of IRS-1 Ser^636/639^ phosphorylation in fibroblasts derived from young (3-6 mos) WT and *Ames* dwarf mice. **C.** A bar graph and **D.** immunoblot analysis of the phosphorylation of IRS-1 Ser^636/639^ in fibroblasts derived from aged (21-24 mos) WT and *Ames* dwarf mice. Statistical analyses were performed using the two-tailed *t*-test as described in Figure 1 and in METHODS.

The Ser^636/639^ residues are involved in the regulation of protein synthesis (mTOR, S6K, ERK) and degradation [28]. Our data show a strong stimulation of Ser^636/639^ phosphorylation in young WT mice whereas it is virtually non-responsive in dwarf fibroblasts (Figure 3A and 3B). Thus, the *Ames* fibroblasts are refractory to IGF-1 stimulated phosphorylation of Ser^636/639^ which could result in the reduced mTOR/S6K1 signaling, attenuation of protein synthesis and decreased insulin resistance. This is a characteristic of longevity in models ranging from nematodes to mice. On the other hand Ser^636/639^ phosphorylation is strongly induced by IGF-1 in both aged WT and *Ames* fibroblasts which is a characteristic of insulin resistance (Figure 3C and 3D). The phosphorylation in the dwarf fibroblasts decreases significantly at 30 minutes and 60 minutes whereas such a decrease is delayed in WT cells. The sustained vs. transient phosphorylation of Ser^636/639^ is indicative of differences in metabolic characteristics such as protein synthesis and insulin resistance.

### IGF-1 stimulates Ser^1101^ phosphorylation in young and aged wild type and aged *Ames* dwarf fibroblasts

Infusion of amino acids into humans leads to the activation of S6K1 phosphorylation of IRS-1 Ser^1101^, a reduction of IRS-1 function and insulin resistance in skeletal muscle [29]. Mutation of this site blocks the ability of amino acids to suppress IRS-1 Tyr and Akt phosphorylation [29]. Phosphorylation of IRS-1 Ser^1101^ is increased in livers of obese *db/db* and WT, but not S6K^(−/−)^ mice maintained on a high protein diet. Nutrient dependent activation of S6K1 is thus associated with insulin resistance in mice and humans in part via Ser^1101^ phosphorylation [29].

**Figure 4:**
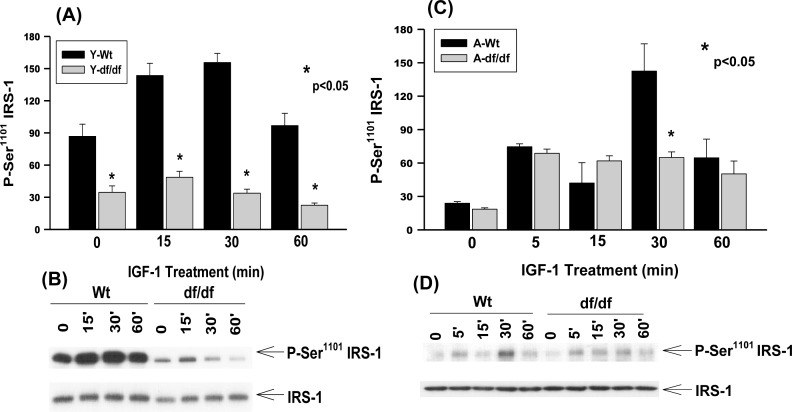
The IGF-1 stimulated phosphorylation of IRS-1 Ser^1101^ in young and aged WT and *Ames* dwarf mouse tail fibroblasts **A.** A bar graph and **B.** immunoblot analysis of the phosphorylation of IRS-1 Ser^1101^ in fibroblasts derived from young (3-6 mos) WT and *Ames* dwarf mice. **C.** A bar graph and **D.** immunoblot analysis of the phosphorylation of IRS-1 Ser^1101^ in fibroblasts derived from aged (21-24 mos) WT and *Ames* dwarf mice. Statistical analyses were performed using the two-tailed *t*-test as described in Figure 1 and in METHODS.

Young WT fibroblasts exhibit a significant level of endogenous phosphorylation of Ser^1101^ and treatment with IGF-1 results in further increased phosphorylation at this site (Figure 4A, 4B). The peak of phosphorylation occurs ~15 minutes after treatment and decreases by ~60 minutes to the level of untreated cells suggesting a rapid response to and recovery from IGF-1 treatment in young WT fibroblasts.

The endogenous level of Ser^1101^ phosphorylation in young *Ames* mice is ~3-fold lower than the corresponding untreated WT cells (Figure 4A and 4B). Furthermore, IGF-1 treatment of the *Ames* cells showed a slight increase in Ser^1101^ phosphorylation at 15 minutes compared to the WT and at 30- and 60 minutes the level of phosphorylation decreases to the level of untreated cells.

Similar analyses of the aged WT and dwarf fibroblasts show that the endogenous levels of Ser^1101^ phosphorylation are significantly lower compared to the young cells, *i.e.,* ~3-fold in WT and ~2-fold in dwarf cells (Figure 4C and 4D). The IGF-1 treated WT and dwarf cells showed a similar response after 5 and 15 minutes of treatment; at 30 minutes, however, the phosphorylation was ~5-fold higher than its endogenous level which is the same as that seen in the young WT fibroblasts. These data indicate that the induction of IGF-1 phosphorylation of Ser^1101^ is delayed in the WT aged cells. Furthermore, there is a rapid loss of P-Ser^1101^ by 60 minutes so that the level of phosphorylation by the WT fibroblasts is the same as in the *Ames* cells. On the other hand there is also a lag period in response to IGF-1 in the aged WT fibroblasts that does not occur in the young cells.

## DISCUSSION

Using genetically identical young and aged WT and *Ames* dwarf fibroblasts we have identified specific Ser residues of IRS-1 that are substrates for IGF-1 stimulated phosphorylations and are the same as those for insulin stimulated Ser phosphorylation. We thus propose that this may be a crosstalk mechanism by which circulating IGF-1 participates in the regulation of insulin sensitivity and plays an important role in the hormonal balance between GH and insulin thus enabling them to regulate the same metabolic pathways [4]. Importantly, the levels of endogenous and inducible phosphorylation of Ser^307^, Ser^612^, Ser^636/639^, and Ser^1101^ are significantly higher in the WT fibroblasts suggesting that these epigenetic alterations in the *Ames* mice control the unique serine phosphorylation patterns that determine the physiological conditions of IRS-1 signaling associated with longevity. We thus propose that epigenetic changes associated with the hormonal deficiencies of the *Prop1^(−/−)^* mutation are stabilized and maintained in fibroblasts derived from these mice [23, 30, 31]

The IGF-1 mediated phosphorylation of IRS-1 Ser^307^, Ser^612^, Ser^636/639^, and Ser^1101^ is attenuated in *Ames* cells. Since these phosphorylations regulate multiple signaling pathways and metabolic processes the overall reduced levels of phosphorylation will, for example, affect levels of protein synthesis, fatty acid metabolism, glucose metabolism and stress response activity characteristic of the long-lived *Ames* mice [30, 31]. This is consistent with the reported specificities of Ser phosphorylation that include both normal and disease-associated metabolic functions. An interesting example is the phosphorylation of Ser^612^ which is virtually absent in the fibroblasts from young *Ames* mice. Since phosphorylation of IRS-1 Ser^612^ is a physiological characteristic of cardiovascular pathology [25, 32], type 2 diabetes [26], obesity and hypertension [26, 27], our data suggest that the high levels of phosphorylation of Ser^612^ in young WT mice may promote cardiovascular pathology and also serve as a marker of this disease as well as of aging. Alternatively, the virtual absence of Ser^612^ phosphorylation in young *Ames* fibroblasts and rapid loss of P-Ser^612^-IRS-1 by aged *Ames* fibroblasts suggests a decreased risk of cardiovascular disease associated with longevity. The fact that phosphorylation levels are significantly elevated in fibroblasts from aged WT and dwarfs suggest that this increased endogenous activity and strong stimulation of phosphorylation may represent major age-associated changes in the IRS-1 targeted metabolic functions.

There is a significant difference between the induction of Ser^307^, Ser^612^, and Ser^636/639^ phosphorylation in aged WT *vs.* aged *Ames* fibroblasts. For example, the level of phosphorylation in aged WT fibroblasts remains elevated up to 60 minutes whereas the phosphorylation declines after 15-30 minutes in the age-matched *Ames* fibroblasts. Thus, the WT-specific, sustained and elevated level of phosphorylated IRS-1 Ser residues may be part of the mechanism of inhibition of IRS-1 activity associated with resistance to insulin/IRS-1. On the other hand, although the *Ames* fibroblasts respond strongly to IGF-1, the decrease of Ser phosphorylation suggests this rapid recovery may be the mechanism that protects against the consequences of IRS-1 hyperphosphorylation.

The insulin/IGF-1 and mammalian mTOR pathways are highly conserved physiological processes that protect against the development of the aging phenotype. The multiple physiological consequences of IRS-1 phosphorylations are seen in increased activation of the mTOR pathway along with enhanced phosphorylation of IRS-1 at Ser^307^ and Ser^636/639^ that occur in animal models of insulin resistance [30]. Thus, activation of the mTOR pathway leading to the down-regulation of IRS-mediated signals is a negative feedback mechanism which may be involved in the development of insulin resistance [33, 34]. Reduced insulin/IGF-1 signaling extends lifespan in both invertebrates and vertebrates [35, 36]. Thus, low levels of insulin and/or IGF-1 signaling (together with high insulin and IGF-1 sensitivity) are physiological characteristics that support extended lifespan of the *Ames* mutants [37]. That deletion of IRS-1 extends lifespan and increases resistance to several age-related pathologies in male and female mice is further support of this mechanism [9, 36]. These studies suggest that reduced IRS-1 dependent signaling is a robust mechanism for the modulation of mammalian lifespan.

It has been shown that the haploinsufficient IGF-1R^(+/−)^ mouse down regulates the principle pathways stimulated by IGF-1 [8]. Embryonic fibroblasts derived from *Igf1r^(+/−)^* mice showed a 50% reduction in IGF-1R levels and a marked reduction in IGF-1 induced tyrosine phosphorylation of its substrate, IRS-1. This suggests that the down-regulation of IGF-1R in haploinsufficient mice down-regulates the principal pathways stimulated by IGF-1 and that the general decrease of IGF-1 via a decrease in IGF-1R can increase mammalian lifespan [8].

Phosphorylation of IRS-1 Ser^307^ exemplifies a common site that integrates heterologous inhibition of insulin signaling by multiple factors (See Ref.^15^ for a detailed Table). For example, Ser^307^ phosphorylation is stimulated by insulin, IGF-1, TNFα [38], anisomycin [36], fatty acids [39], amino acids [10] and C-reactive protein [40]. Our studies suggest that IGF-1 may act synergistically with these factors to promote Ser^307^ phosphorylation [41] and attenuation of IRS-1 and its targeted downstream pathways, *e.g.,* PI3K and MAPK pathways [40, 42].

We propose that crosstalk between insulin and IGF-1 signaling is mediated by the phosphorylation/dephosphorylation of Ser/Thr and Tyr residues, and that this is the mechanism of regulation of the major pathways that target physiological functions associated with longevity determination.

## MATERIALS AND METHODS

### Isolation and treatment of *Ames* mouse tail fibroblasts

The isolation of fibroblasts from young (3-6 mos) and aged (21-24 mos) wild-type and *Ames* dwarf mice has been described [23]. In this study, the fibroblasts at passage 4 or 5 were plated in 100 mm^2^ cell culture dishes (2×10^5^ cells/dish) and cultured for 3 days in DMEM medium containing 15% FBS. The day before treatment, the medium was replaced with a medium containing 0.5% FBS. The recombinant human IGF-1 (Sigma) was prepared in distilled H_2_O and filter sterilized. The IGF-1 stock solution (25 ng/ml) was diluted in DMEM medium containing 0.5% FBS and the fibroblasts were treated with IGF-1 at a final concentration of 2 ng/ml. The cells were harvested and cytoplasmic and nuclear extracts were prepared [23]. The protease and phosphatase inhibitors were added to the extraction buffers prior to use [23]. The protein concentration of the extracts was determined using Bradford reagent (Bio-Rad).

### Western blot analyses and immunoprecipitation assays

Western blot analyses and immunoprecipitation assays were performed as described [23]. Antibodies used for immunoblot analysis of phosphorylated proteins were from Cell Signaling Technology: anti-phospho Ser^307^ IRS-1, anti-phospho Ser^612^ IRS-1, anti-phospho Ser^636/639^ IRS-1 and anti-phospho Ser^1101^ IRS-1. The antibody was from Sigma.

### Statistical analyses

Statistical analyses were performed for age-matched comparisons, the single dependent variable being the *Ames* dwarf mutants. The normalized values of protein and phosphorylated protein bands were analyzed using the 2-tailed *t*-test to test the mean difference between age-matched groups at a significance level of 0.05. The symbols (*) indicate statistical significance for the values represented by the bar or time-point.
